# Failures

**Published:** 1874-12

**Authors:** 


### FAILURES.

"I always learn more by failures than by success."

We have heard this and similar expressions so often in discussions in our societies, that we have recently been trying to think that it is not a falsehood, but it is one of the things that will not look like truth.

Upon investigation we find that pretty much all that we have that is worth knowing, was learned by doing things the right way.

Now it is an easy thing to make failures, and upon the theory of this quotation, there are some dentists we wot of who are, or ought to be, the most learned people in the world for they have made little else than failures—have not been troubled with successes. The advocate of this theory should make nothing but failures, especially if learning were his object. Well this brings things round to a far more encouraging condition than we ever imagined would be attained this side of the mellennium. The way to get instruction is to make failures, it is far easier to make failures than success, and there are a great many more ways to do it too; and variety as everybody knows is the spice of life. We don't see the use after this of making anything but failures, and yet it is probable that there will still be some chuckle heads who will be trying to make success and remain in ignoracee, this will doubtless be the case till all the fools are dead.

It doesn't require much knowledge to make failures. This quotation is an illustration of perpetuating nonsense that somebody has started, for want of something better to say, and it is kept up because of its jingle.

